# Accelerating *Dalbergia odorifera* Plantation Breeding: SSR-Based Genetic Diversity and Trait Associations for Enhanced Heartwood Yield

**DOI:** 10.3390/plants14243787

**Published:** 2025-12-12

**Authors:** Xinyue Hou, Ruxue Bai, Rongtao Li, Jiawen Li, Yun Yang, Haoling Li, Bo Chen, Liangming Huang, Hui Meng, Jianhe Wei

**Affiliations:** 1 Institute of Medicinal Plant Development, Chinese Academy of Medical Sciences and Peking Union Medical College, Beijing 100193, China; houxinyue37@163.com (X.H.);; 2 Hainan Provincial Key Laboratory of Resources Conservation and Development of Southern Medicine and Key Laboratory of State Administration of Traditional Chinese Medicine for Agarwood Sustainable Utilization, Hainan Branch of the Institute of Medicinal Plant Development, Chinese Academy of Medical Sciences and Peking Union Medical College, Haikou 571533, China

**Keywords:** *Dalbergia odorifera* T. Chen, plantations, SSR markers, genetic diversity, phenotype trait association analysis, heartwood ratio (HWR)

## Abstract

*Dalbergia odorifera* T. Chen possesses significant aromatic, medicinal, and timber value, yet its wild populations are critically endangered due to habitat degradation. Breeding programs are urgently needed to address resource shortages, but the suitability of large-scale plantations as alternative genetic resources remains unverified. This study systematically evaluated the genetic diversity of 380 individuals from five populations using 24 polymorphic SSR markers, identifying 278 alleles. The results demonstrated a moderate level of genetic diversity in plantation populations, comparable to wild resources. Additionally, nine phenotypic traits were measured in 70 individuals. Correlation analysis revealed that the heartwood ratio (HWR) was significantly positively correlated with diameter at breast height (DBH) and ground diameter (GD) (*p* ≤ 0.05). Our association analysis, based on general linear (GLM) and mixed linear models (MLM), revealed two key findings: one locus (96c-345) was significantly associated with diameter traits, and four loci (34a-241, S03-265, JXHT097-252, JXHT136-270) were strongly linked to the HWR (*p* ≤ 0.01). This research provides initial evidence that plantations are viable substitutes for wild germplasm and establishes a foundation for marker-assisted breeding in this valuable species.

## 1. Introduction

*Dalbergia odorifera* T. Chen, a large and rare tree species within the genus Dalbergia (Fabaceae family), possesses notable aromatic, medicinal, timber, and cultural value [1,2]. The dried heartwood derived from its trunk and roots constitutes the traditional and highly prized Chinese medicinal material known as “Jiangxiang” [2], which exhibits a wide spectrum of pharmacological properties, including anti-myocardial ischemia [3,4], antioxidant [5,6], and anti-inflammatory [7] properties, among others [8,9,10]. Furthermore, renowned globally for its distinctive fragrance and exquisite grain patterns, *D. odorifera* heartwood ranks among the world’s most precious timbers [11,12]; it is extensively utilized in high-end furniture, intricate handicrafts, prestigious musical instruments, and luxury decorative items [2].

However, due to centuries of over-exploitation and habitat destruction, wild populations of *D. odorifera* are now critically endangered [11,13]. Reflecting this dire situation, the species has been listed as (1) Vulnerable (VU) on the IUCN Red List of threatened Species (World Conservation Monitoring Centre, 1998) [14]; (2) critically endangered (CR) on the China Biodiversity Red List (China Biodiversity Red List, MEE & CAS, 2021) [15]; and (3) a National Grade II Protected Wild Plant Species under the List of National Key Protected Wild Plants (NKPWPL; China’s State Council, Decree No. 15, 2023) [16].

The drastic decline in natural *D. odorifera* resources has led to soaring market prices [17]. To meet market demand and alleviate pressure on wild resources nearing extinction, large-scale plantations have been established in regions beyond its native Hainan Province over the past decade [18,19,20]. However, heartwood formation in *D. odorifera* is protracted and complex. Previous studies indicate that the species typically requires 6–8 years to initiate heartwood development and must grow for over 30 years to attain marginally viable economic value [2,21].

To accelerate heartwood formation, various artificial induction techniques have been developed, including physical wounding [22], chemical induction [17], phytohormone treatment [23,24,25,26], fungal induction [27], and silvicultural interventions [28,29,30,31]. However, induced heartwood currently fails to fully substitute for the quality derived from wild trees [20]. Moreover, most plantation stocks originate from unselected seeds, and our unpublished data indicate a low heartwood formation rate (~10% in 20-year-old trees), underscoring the urgent need for advanced breeding.

Confronting this resource bottleneck, the fundamental question remains as to whether large-scale plantations can functionally substitute for wild germplasm as the genetic base for breeding. Field studies reveal that the present cultivation system relies heavily on a narrow genetic base, originating from a limited number of wild trees transplanted decades ago. The continued propagation of these founder trees via seeds and cuttings has resulted in low genetic diversity within plantation populations. Over time, this limited genetic diversity, coupled with the absence of specialized breeding programs, has led to trait instability. Assessing the genetic diversity of these plantation resources is therefore a critical first step. Simple sequence repeats (SSRs) have proven to be a robust tool for such genetic assessments and for the association mapping of important traits in plants.

While SSR markers have been used to analyze genetic diversity in limited wild *D. odorifera* samples [32,33], a systematic evaluation of plantation populations and association analysis for key economic traits are still lacking. Therefore, this study aims to (1) assess the breeding potential of major *D. odorifera* plantation populations by evaluating their genetic diversity and (2) identify SSR markers associated with growth and heartwood traits through association analysis. The findings will provide crucial evidence for utilizing plantation resources and advancing marker-assisted breeding in this endangered species.

## 2. Results

### 2.1. Genetic Diversity and Population Structure of D. odorifera Plantations

As Table 1 shows, a total of 24 polymorphic SSR markers were genotyped across 380 *Dalbergia odorifera* T. Chen (*D. odorifera)* individuals. Among these, four markers were novel primers developed by our research group, and 20 markers were previously published primers.

As detailed in Table 2, the 24 polymorphic SSR primers successfully amplified in 365–380 individuals (mean N = 376.5), confirming high amplification efficiency. Collectively, these markers detected 128 alleles, with per-locus observed alleles (*N_a_*) ranging from 2 to 12 (mean = 5.3) and effective alleles (*N_e_*) from 1.391 to 6.543 (mean = 2.5144). Genetic diversity analysis revealed the following: Shannon’s index (*I*) ranged from 0.546 to 2.023 (mean = 1.0301), indicating moderate species-level diversity. Observed heterozygosity (*H_o_*) ranged from 0.274 to 0.712 (mean = 0.5175), and expected heterozygosity (*H_e_*) ranged from 0.281 to 0.847 (mean = 0.5446), demonstrating adherence to the Hardy–Weinberg equilibrium (*H_o_* ≈ *H_e_*) and moderate to high population diversity (both >0.5). The fixation index (*F*) ranged from −0.061–0.327 (mean = 0.0409), suggesting slight heterozygote deficiency but no severe inbreeding. Intrapopulation gene diversity (*H_s_*) ranged from 0.639 to 0.884 (mean = 0.7517), reflecting high within-population variation. Polymorphism information content (*PIC*) ranged from 0.269 to 0.829 (mean = 0.4925), classified as moderate polymorphism (0.25 ≤ *PIC* ≤ 0.5) and high polymorphism (*PIC* ≥ 0.5), indicating moderate to high population diversity. Population structure metrics showed the following: Genetic differentiation (*F_st_*) ranged from 0.013 to 0.212 (mean = 0.0611), indicating moderate differentiation. Gene flow (*N_m_*) ranged from 0.931 to 19.458 (mean = 6.3438), implying frequent gene exchange and weak geographical isolation. *G_st_* ranged from 0.004 to 0.205 (mean = 0.0532), reinforcing moderate species-wide diversity in *D. odorifera*.

**Figure 1:**
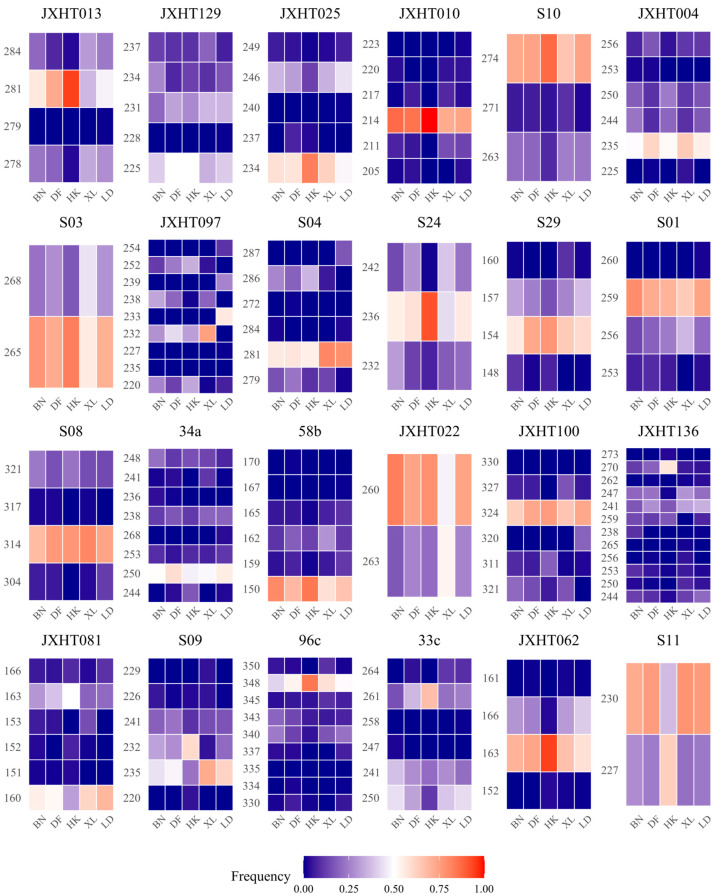
A frequency heatmap of alleles at 24 SSR loci in five populations. The *X*-axis represents the five populations sampled in this study, and the *Y*-axis represents different alleles. The intensity of the red color indicates a higher frequency of the allele within the corresponding population samples, whereas deeper blue signifies a lower frequency.

Genetic diversity statistics for five sampling locations are detailed in Table 3 and Figure 1. The key findings include the following: Observed alleles (*N_a_*) ranged from 3.458 to 4.458 (mean = 4.0500). Effective alleles (*N_e_*) spanned 1.888–2.519 (mean = 2.2556). Shannon’s index (*I*) ranged from 0.734 to 0.998 (overall mean = 0.9187), confirming moderate genetic diversity. Heterozygosity values were as follows: Observed heterozygosity (*H_o_*) ranged from 0.354 to 0.557 (overall mean = 0.4840). Expected heterozygosity (*H_e_*) ranged from 0.402 to 0.540 (mean = 0.5035). Unbiased expected heterozygosity (*uH_e_*) ranged from 0.408 to 0.542. All three heterozygosity indices clustered near 0.5, reinforcing moderate genetic diversity. The fixation index (*F*) ranged from −0.034 to 0.098 (mean = 0.0372), indicating slight heterozygote deficiencies. Notably, Haikou (HK) exhibited the lowest values for *N_a_, N_e_, I*, *H_o_*, *H_e_*, *uH_e_*, and *F*, while Xishuangbanna (BN) showed the highest values across all parameters. This pattern likely reflects sampling size effects: BN had the largest sample size (enhancing diversity detection), whereas HK had the smallest (constraining variation estimates).

**Figure 2:**
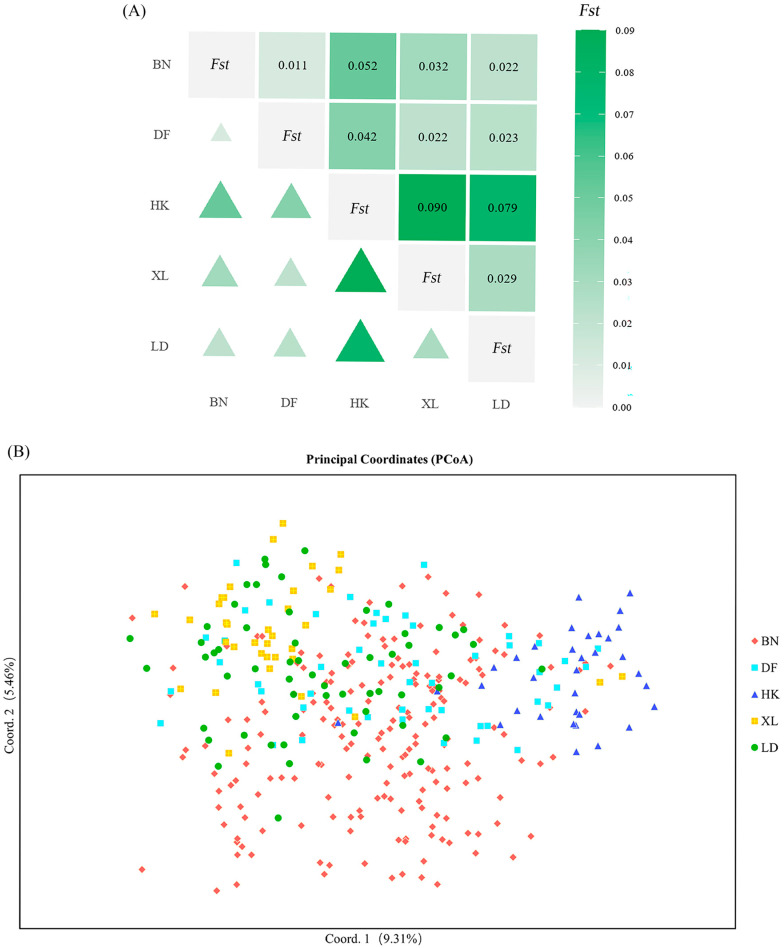
Differentiation analysis across populations. (**A**) Pairwise *F_st_* values among five populations. Upper triangle: *F_st_* value; lower triangle: degree of differentiation between populations. The intensity of the green color indicates the degree of differentiation between groups. The deeper the color, the larger the data, and the larger the triangle, the higher the degree of differentiation between groups. (**B**) A two-dimensional plot of the principal coordinate analysis (PCoA) of 380 individuals.

Pairwise analyses among BN, XL, HK, LD, and DF populations (Figure S1 and Figure 2A) demonstrated that genetic differentiation coefficients (*F_st_*) range theoretically from 0 (undifferentiated populations) to 1 (complete allelic fixation). The *F_st_* of the HK population and the BN, XL, and DF populations is greater than 0.05 but less than 0.15, which means that there is a certain degree of differentiation between these populations. Nei’s genetic distance analysis (*D* value) between populations is similar to genetic differentiation analysis; that is, there is a certain degree of differentiation between the HK population and the BN, XL, and DF populations. However, the *D* value of the DF, BN, and XL populations are low, while their *I* (Nei’ genetic identity) values are high, which means that these populations may originate from the same region. Conseversely, the low *I* values between the HK population and the LD, XL, and DF populations indicate substantial differentiation. These results from *D* and *I* values are consistent with the genetic differentiation analysis revealed by the pairwise *F_st_* above.

A molecular analysis of variance (AMOVA) showed (Table 4) that 5.39% of the genetic variation was caused by differences between populations, including 199 BN individuals, 36 XL individuals, 35 HK individuals, 50 DF individuals, and 60 LD individuals. Meanwhile, the remaining 3.52% and 91.09% of the genetic variation were found to occur between individuals and within individuals, respectively. The small percentage of variance between populations and between individuals within populations indicates that genetic variation mainly originates from within populations, with weak differentiation between populations and possible problems such as inbreeding and weak genetic drift within populations. The intra-individual variance accounted for a large proportion of the variance, a common pattern in diploid species.

A two-dimensional plot of the principal coordinate analysis (PCoA) (Figure 2B) was derived from the first two components of the genotype data for 380 *D. odorifera* individuals. It revealed the genetic relationships among individuals and populations, with the first two components explaining 9.31% and 5.46% of the total variation, respectively. The plot showed that the distribution of the HK, XL, and LD groups was more concentrated, while the distribution of the BN and DF groups was scattered, and the variability between the two groups was consistent with the previous genetic differentiation between the two groups.

**Figure 3:**
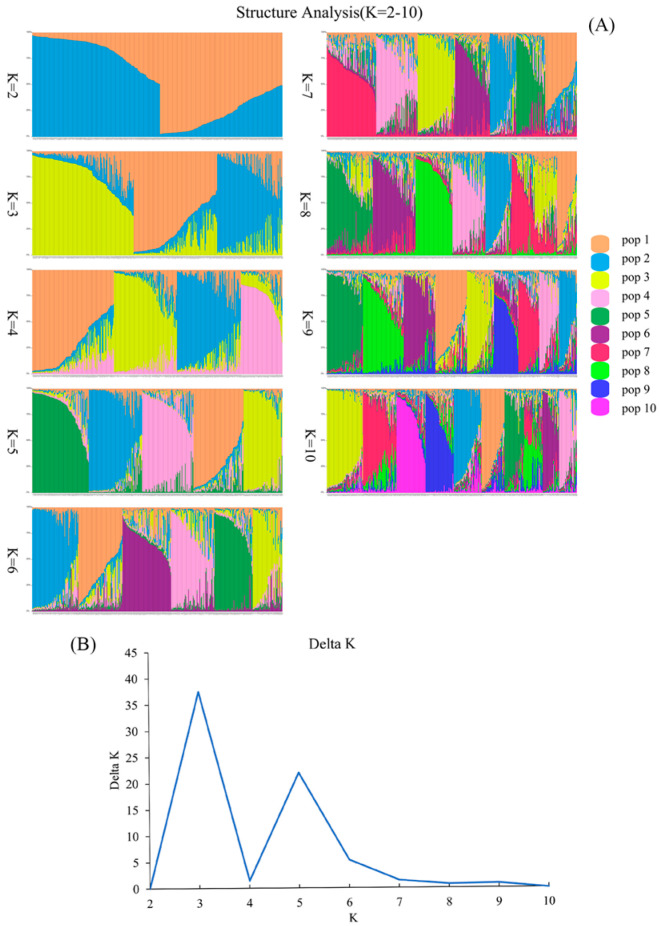
Population structure analysis. (**A**) Population structure analysis, depicted with each column representing an individual, where the length of the different color segments signifies the proportion of an ancestor. The parameter K = 2–10 denotes the number of ancestral groups assumed in this study, ranging from 2 to 10. (**B**) The SSR-based determination of the optimal number of subpopulations in the studied genotypes of *D. odorifera* (K value).

In order to analyze the population structure and determine the actual number of subpopulations, the optimal number of subpopulations was calculated based on the variation in Δ K with the value of K using STRUCTURE v2.3.4 software. The results showed that the inferred number of subpopulations was three in the studied genotypes of *D. odorifera* (Figure 3A,B). Therefore, K = 3 was considered the optimal number of subpopulations for analyzing the population structure.

### 2.2. Cross-Amplification of SSR Markers to Other Species

**Figure 4:**
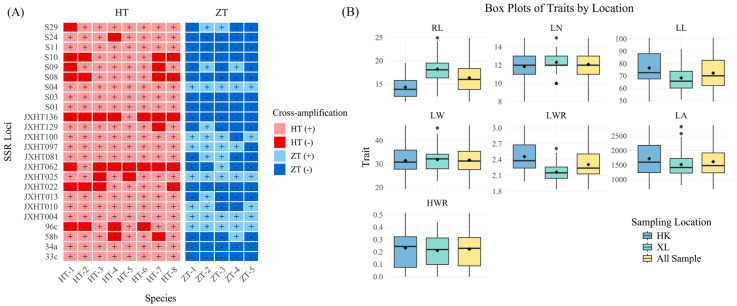
Cross-amplification of SSR markers to other species and analysis of phenotypic traits. (**A**) Cross-amplification of 24 pairs of polymorphic SSR primers in eight species of *Dalbergia L. f.* and five species of *Pterocarpus Jacq.* HT: *Dalbergia L. f.*; ZT: *Pterocarpus Jacq*; HT-1: *Dalbergia oliveri* Gamble ex Prain; HT-2: *Dalbergia bariensis* Pierre; HT-3: *Dalbergia cultrate* Graham ex Benth.; HT-4: *Dalbergia benthamii* Prain; HT-5: *Dalbergia sissoo* Roxb. ex DC.; HT-6: *Dalbergia cochinchinensis* Laness; HT-7: *Dalbergia balansae* Prain; HT-8: *Dalbergia hainanensis* Merr. & Chun; ZT-1: *Pterocarpus marsupium* Roxb.; ZT-2: *Pterocarpus macrocarpus* Kurz; ZT-3: *Pterocarpus septentrionalis* Donn. Smith; ZT-4: *Pterocarpus indicus* Wall.; ZT-5: *Pterocarpus echinatus* Pers. ‘+’ indicates that SSR loci were all successfully transferred, and ‘-’ indicates that SSR loci were not amplified successfully. (**B**) Box plots display performance of traits studied for HK, XL, and all samples. DBH: Diameter at Breast Height; GD: Ground Diameter. RL: Rachis Length; LN: Leaf Number; LL: Leaf Length; LW: Leaf Width; LWR: Length–Width Ratio; LA: Leaf Area; HWR: Heartwood Ratio.

**Figure 5:**
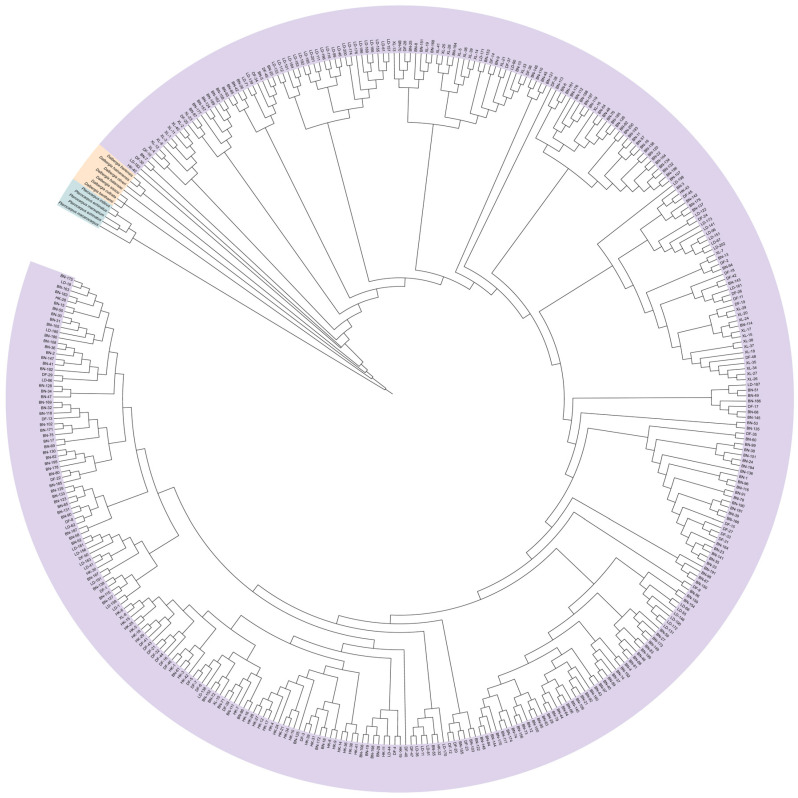
Construction of NJ trees based on Nei’s unbiased genetic distances for five populations of *D. odorifera* and outgroup species.

SSR markers are commonly used for the identification of species genetic diversity and for species identification and the differentiation of related species. To investigate whether the 24 pairs of polymorphic SSR markers used in this study are species-specific, we cross-amplified eight species of *Dalbergia* L. f. (*Dalbergia oliveri* Gamble ex Prain, *Dalbergia bariensis* Pierre, *Dalbergia cultrate* Graham ex Benth., *Dalbergia benthamii* Prain, *Dalbergia sissoo* Roxb. ex DC., *Dalbergia cochinchinensis* Laness., *Dalbergia balansae* Prain, *Dalbergia hainanensis* Merr. & Chun) and five species of *Pterocarpus* Jacq. (*Pterocarpus marsupium* Roxb., *Pterocarpus macrocarpus* Kurz, *Pterocarpus septentrionalis* Donn. Smith, *Pterocarpus indicus* Wall., *Pterocarpus echinatus* Pers.). In Figure 4A and Table S1, it was found that most SSR loci did not differentiate species of *Dalbergia* L. f. from *D. odorifera* well, and only two markers, JXHT136 and JXHT062, had low amplification rates (12.5%), which denotes high differentiation. Most of the SSR loci were able to distinguish the species of *Pterocarpus* Jacq. from *D. odorifera* well. Ten markers, S10, S03, S24, S01, S08, 34a, JXHT022, JXHT136, 33c, and S11, had 0% amplification and were able to distinguish the species of *Pterocarpus* Jacq. from *D. odorifera* completely. As for JXHT025, JXHT004, S04, and 96c, these four markers showed an amplification rate of 100% in *Pterocarpus* Jacq., indicating no discriminatory power based on the presence/absence of amplification for distinguishing between *Pterocarpus* and *D. odorifera*.; JXHT136 is the best at distinguishing the species of the outer population. Evolutionary trees of the outgroups and five populations of *D. odorifera* were constructed using the neighbor-joining (NJ) method (Figure 5). The results showed that 380 samples of *D. odorifera* were able to cluster into one large clade, and the five species of *Pterocarpus* Jacq. were able to cluster into one small clade, and all of them were distinguished from the eight species of *Dalbergia* L. f.

### 2.3. Phenotypic Variation and Correlations

**Figure 6:**
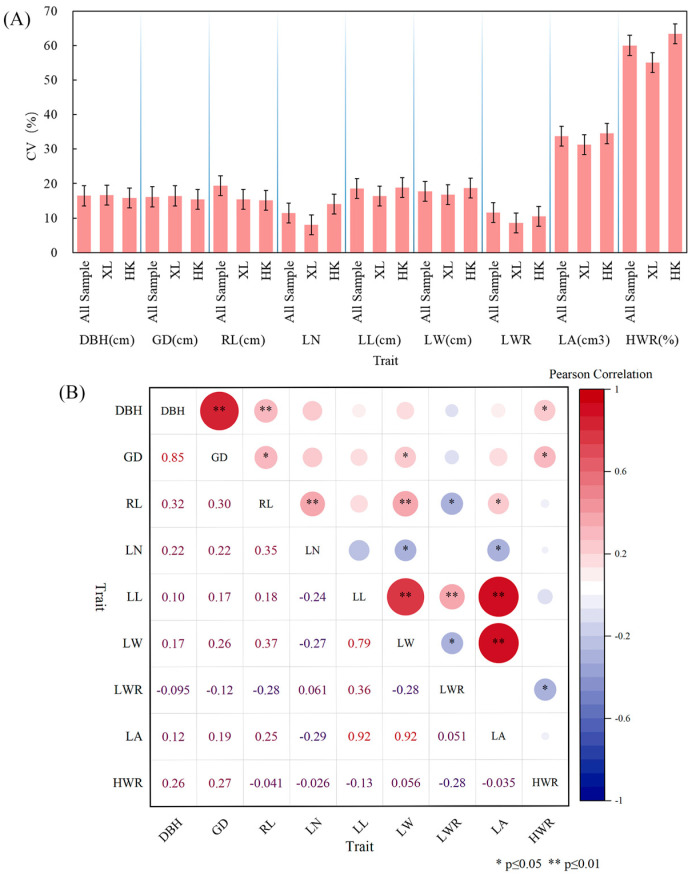
Phenotypic variation and correlations. (**A**) Visualization of coefficient of variation (*CV*) for nine phenotypic traits among different populations. (**B**) Correlation analysis between pairwise phenotypic traits. Lower triangle: correlation coefficient; upper triangle: significance. *: *p* ≤ 0.05; **: *p* ≤ 0.01.

Figure 4B displays the performance of the traits studied for HK, XL, and all samples. According to the box plot, most of the traits displayed similar patterns across the locations except for Rachis Length (RL), Leaf Length (LL), and Leaf Length-width Ratio (LWR). The coefficient of variation (*CV*) is a relative measure of the degree of dispersion between phenotypic data, which eliminates unit and mean differences and facilitates comparisons of the degree of variation between different samples or populations. Therefore, *CV* was calculated for the phenotypic traits of different populations (Table 5 and Table S2, Figure 6A), and the average *CV* for the nine phenotypic traits of all samples was 22.78%, the largest *CV* was seen for the Heartwood Ratio (HWR) (60.04%), and the smallest was seen for Leaf Number (LN) (11.40%). Both for all samples and for each population, it can be seen that the HWR showed a high coefficient of variation, indicating considerable phenotypic diversity and genetic instability; diameter at breast height (DBH) and ground diameter (GD) had a medium coefficient of variation and high phenotypic diversity; and for the leaf-related indices (RL, LN, LL, LW, LWR, and LA), all except leaf area (LA) exhibited a relatively low coefficient of variation (*CV*), indicating low phenotypic diversity and relatively stable expression of these traits. This pattern was particularly evident for the leaf width ratio (LWR).. The mean *CV*s of XL and HK were 20.50% and 22.90%, respectively, which were not much different from those of the overall samples, indicating that there was not much difference in phenotypic diversity among different groups.

The correlations between pairwise phenotypic traits are shown in Figure 6B. Highly significant positive correlations (*p* ≤ 0.01) were found between the following trait pairs: DBH and GD; DBH and RL; RL and LN; RL and LW; LL and LW; LL and LWR; LL and LA; and LW and LA. DBH and HWR; GD and RL, LW, HWR; and RL and LA all showed significant positive correlations (*p* ≤ 0.05). Significant negative correlations (*p* ≤ 0.05) were found between: RL and LWR; LN and LW; LN and LA; LW and LWR; and LWR and HWR.

### 2.4. Trait–Marker Association

**Figure 7:**
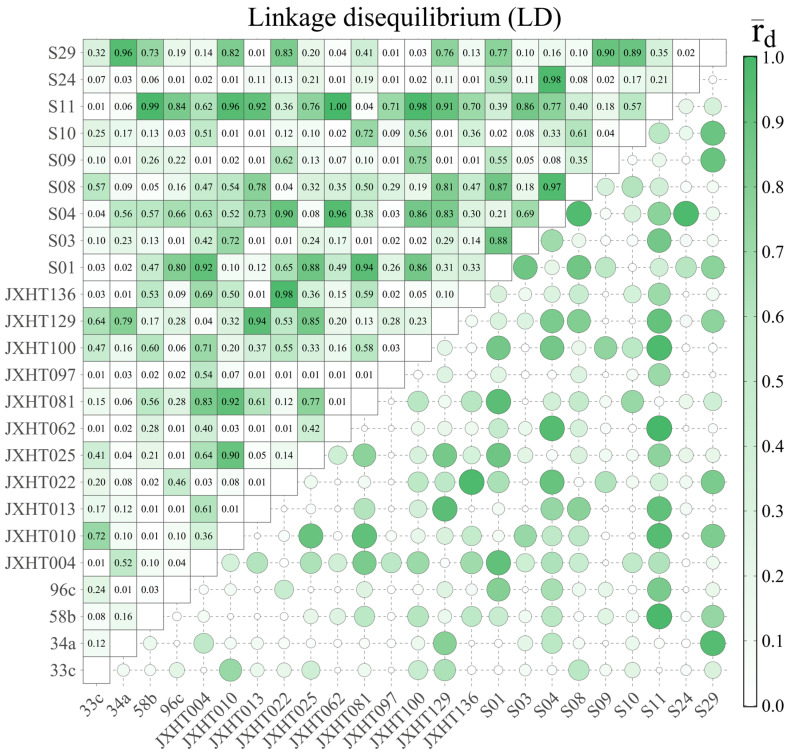
Linkage disequilibrium (LD) analysis of 24 pairs of SSR markers.

Linkage disequilibrium (LD) is the result of the non-random association of alleles on the same chromosome or at different loci between different chromosomes within a population. The higher the level of LD, the tighter the linkage. A total of 276 pairs of SSR loci were combined in the LD analysis of 24 SSR loci (Figure 7). Under the threshold of *p* ≤ 0.01, 97 pairs of SSR loci were found to have significant linkage disequilibrium, accounting for 35.14% of all locus combinations. In total, 111 pairs of SSR loci were found to have rD ≥ 0.3 (strong LD), accounting for 40.22% of all locus combinations, and 168 pairs of SSR loci were found to have rD ≥ 0.1 (moderate LD), accounting for 60.87% of all locus combinations. The feasibility of trait association depends on the low LD level, and most of the pairs of SSR loci used in this study are in linkage equilibrium, which means that these markers are suitable for the analysis of SSR association with phenotypic traits in the 70 *D. odorifera* individuals.

**Figure 8:**
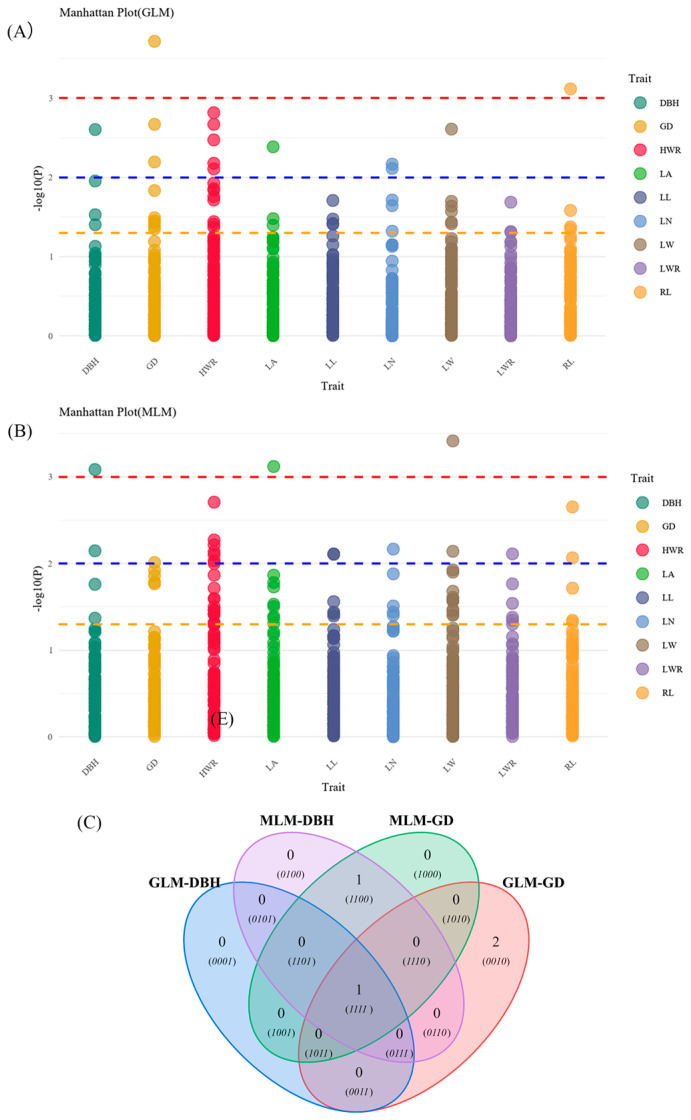
Manhattan plots and Venn diagram of association analysis results. Manhattan plots for General linear model (GLM) (**A**) and Mixed linear model (MLM) (**B**) of studied traits. Yellow, blue, and red dashed lines represent *p* = 0.05, 0.01, and 0.001 thresholds, respectively. (**C**) Venn diagram; GLM-DBH: results significantly associated with DBH using GLM; MLM-DBH: results significantly associated with DBH using MLM; GLM-GD: results significantly associated with GD using GLM; MLM-GD: results significantly associated with GD using MLM; (*0010*): JXHT136-250, S01-256; (*1100*): 96c-340; (*1111*): 96c-345.

**Figure 9:**
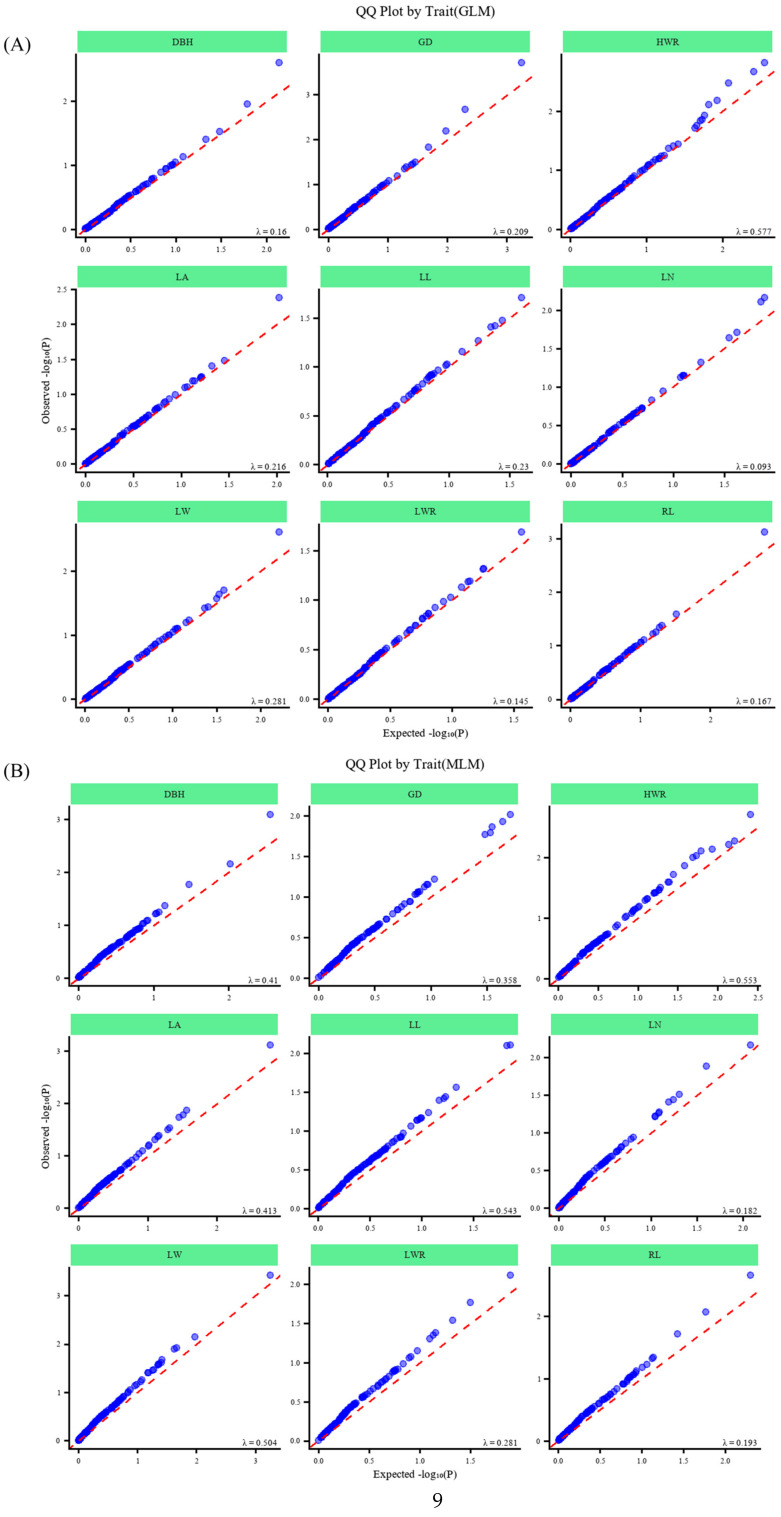
QQ plots of association analysis results. QQ plots for GLM (**A**) and MLM (**B**) of studied traits.

The results of association analysis with phenotypic traits are shown in Table 6 and Figure 8 and Figure 9. General linear model (GLM) analysis showed that eight traits were significantly associated with 16 allelic loci of SSRs (*p* ≤ 0.05), of which seven traits were highly significantly associated with 12 SSR loci (*p* ≤ 0.01), and the variation in the explained rate (R^2^) ranged from 3.85% (RL and JXHT136-253) to 18.97% (GD and 96c-345). Mixed linear model (MLM) analysis showed that nine traits were significantly associated with 17 allelic loci of SSRs, including a highly significant association with 15 loci, and the variation in the explained rate (R^2^) was 6.69% (HWR and 96c-340) ~ 21.41% (LW and 96c-348). Intersecting the results of the two modeling analyses, we found that a total of four traits were significantly associated with seven SSR loci: DBH with 96c-345; RL with 58b-159; LN with S09-220; and HWR with four loci (34a-241, JXHT097-252, JXHT136-270, and S03-265). The associations of HWR with JXHT097-252, JXHT136-270, and S03-265 were highly significant. In addition, seven traits showed more significant associations with nine SSR loci (one model showed highly significant associations, and one model showed significant associations).

**Table 6:** SSR loci analyzed for traits in 70 individuals using GLM and MLM.

Trait	SSR Marker	GLM			MLM		
		*p*-Value	R^2^ (%)	Significance	*p*-Value	R^2^ (%)	Significance
DBH	96c-340				0.001	17.95	**
	96c-345	0.002	12.45	**	0.007	11.26	**
GD	96c-345	0.000	18.97	**	0.014	9.50	*
	JXHT136-250	0.006	10.66	**			
	S01-256	0.002	13.29	**			
	96c-340				0.010	10.52	**
RL	58b-159	0.001	9.95	**	0.002	15.62	**
	JXHT136-253	0.042	3.85	*	0.009	11.29	**
LN	JXHT136-259	0.008	10.53	**	0.013	10.01	*
	S09-220	0.007	10.85	**	0.007	12.04	**
LL	96c-348				0.008	11.99	**
	S04-279	0.038	6.10	*	0.008	11.94	**
LW	96c-348				0.000	21.41	**
	96c-350				0.007	11.70	**
	S04-286	0.002	13.14	**	0.036	7.00	*
LWR	S09-220				0.008	10.10	**
LA	S04-286	0.004	11.75	**	0.031	7.58	*
	96c-348				0.001	19.65	**
HWR	34a-241	0.002	13.50	**	0.010	10.57	**
	JXHT004-244	0.012	9.30	*	0.002	15.67	**
	JXHT097-252	0.002	14.34	**	0.007	11.51	**
	JXHT136-270	0.003	12.39	**	0.005	12.48	**
	S03-265	0.007	10.74	**	0.006	12.09	**
	S08-314				0.008	11.28	**
	S10-271	0.018	8.33	*	0.009	10.78	**
	96c-345	0.008	10.34	**	0.039	6.69	*

Note: SSR loci that were highly significantly associated with phenotypic traits at the detection threshold of *p* ≤ 0.01 are listed in the table, and if one of the model association analyses resulted in a highly significant association and the other resulted in a significant association (*p* ≤ 0.05), then they are listed together in the table, and if they are left blank, it means that they are not significantly associated (*p* > 0.05). *: *p* ≤ 0.05; **: *p* ≤ 0.01.

Additionally, the analysis (Figure 8C) found that loci 58b-159 and RL, 34-241, JXHT097-252, JXHT136-270, and S03-265 with the HWR showed one-to-one highly significant correlation in the results of the GLM and MLM analyses, and the rest of the loci were significantly associated with multiple traits. And notably, 96c-345 was significantly associated only with DBH, GD, and HWR.

## 3. Discussion

### 3.1. Genetic Diversity and Conservation Implications of Dalbergia odorifera T. Chen Plantations

According to the critically endangered status of *Dalbergia odorifera* T. Chen, driven by decades of over-exploitation and habitat destruction, extensive research efforts have focused on inducing heartwood formation artificially, and large-scale plantations have been established across multiple Chinese provinces to alleviate resource depletion [34]. However, so far, induced timber cannot substitute for the quality from natural trees [20], and the low timber yield efficiency in plantations remains a key constraint, necessitating accelerated breeding initiatives. Compounding these challenges, the near extinction of wild populations, protracted growth cycles, complex genetic mechanisms governing heartwood formation, and the scarcity of high-quality heartwood collectively impede conventional breeding approaches. Consequently, the acquisition of superior germplasm resources constitutes a fundamental prerequisite for genetic improvement programs in this species.

Genetic diversity is a key determinant of breeding potential [35]. Simple sequence repeats (SSRs) have been widely used as robust molecular markers for such assessments due to their high polymorphism and co-dominant nature [36]. This technology has been employed as a robust tool for assessing genetic diversity in *D. odorifera*. However, it remains uncertain whether SSR-based analysis provides a reliable assessment of the potential for large-scale cultivated germplasm to functionally replace wild resources in breeding.

To address this, we employed 24 polymorphic SSR markers to genotype 380 individuals from five plantation populations. Our analysis revealed moderate genetic diversity across the plantations (mean *H_e_* = 0.5446, *PIC* = 0.4925), low genetic differentiation among populations (*F_st_* = 0.0611), and a minor heterozygote deficiency (*F* = 0.0409) likely attributable to historical large-scale logging and subsequent inbreeding among residual trees.

Liu et al. [13] similarly reported moderate genetic diversity in wild *D. odorifera* populations, yet the genetic diversity parameters of our plantation populations were comparable to or higher than those reported for the wild population: observed heterozygosity *H_o_* = 0.28, expected heterozygosity *H_e_* = 0.37, and polymorphism information content *PIC* = 0.36. Conversely, their higher inbreeding coefficient (*F* = 0.16) and genetic differentiation (*F_st_* = 0.11) indicate more severe heterozygote deficiency, which is likely attributable to the serious destruction of wild stands and/or methodological differences. In contrast, Xu et al. [33] documented values closely aligned with our findings, *I* = 1.179, *H_o_* = 0.521, *H_e_* = 0.593, and *PIC* = 0.541, reinforcing the reliability of our assessment. This direct comparison strongly supported the idea that these cultivated resources retained genetic diversity equivalent to wild germplasm, validating their suitability for breeding programs.

Genetic diversity analysis across five geographically distinct populations revealed interpopulation variation in diversity parameters, but despite being a relatively low genetic diversity Haikou (HK) population, it did not deviate from the conclusion that genetic diversity was moderate. A pairwise population genetic differentiation analysis of the different populations revealed that the genetic differentiation between Dongfang (DF) and the Xishuangbanna (BN) and Xinglong (XL) populations was the smallest, which aligns with provenance records of the BN sample that showed that the trees originated from Dongfang City, Hainan Province, whereas the HK population was genetically differentiated from the BN, XL, and DF populations to a certain extent, and it is known that the HK population originated from the progeny of the successful introductions of these trees into the Fujian Province of China, which was introduced from Hainan in the 1950s [37]. The AMOVA showed that the genetic variation in *D. odorifera* mainly originated from individuals within populations (94.61%), and the genetic differentiation between populations was weak. This conclusion aligns with Liu, F. M., and Xu, J. R. did not report comparable AMOVA results.

Given that SSRs are officially endorsed by the International Union for the Protection of New Varieties of Plants (UPOV) as the primary molecular marker system for plant variety protection and DNA fingerprinting [38], we assessed the species specificity of our 24 SSR primers using eight species of *Dalbergia* L. f. and five species of *Pterocarpus* Jacq. The results showed that the SSR primers used in the present study confirmed strong species specificity, validating SSR efficacy for germplasm authentication.

Overall, the above findings demonstrate that, given the scarcity of endangered wild resources, cultivated *D. odorifera* resources can serve as suitable genetic substitutes for breeding efforts. Furthermore, the SSR markers used in this study show strong discriminatory power against related species, making them effective tools for *D. odorifera* identification and authenticity verification.

### 3.2. Trait Variation and Trait–Marker Association in D. odorifera

Phenotypic traits represent observable outcomes of genotype-by-environment interactions. Quantifying their variability provides preliminary insights into underlying genetic diversity and informs selection strategies for economically critical traits (e.g., heartwood formation).

In this study, we quantified nine phenotypic traits across 70 plantation trees (XL and HK populations) sampled from genetic diversity cohorts. The heartwood ratio (HWR), the key economic trait, exhibited the highest phenotypic plasticity with coefficients of variation (*CV*) exceeding 55% in both pooled and population-specific analyses. This indicates substantial genetic heterogeneity in heartwood formation, which is a key trait for pharmaceutical and timber industries. The moderate *CV*s observed for diameter at breast height (DBH) and ground diameter (GD) correspond to high growth-related diversity, whereas most leaf traits were shown to be relatively stable. Critically, the XL and HK populations did not differ much from the overall sample, indicating that there was little difference in phenotypic diversity among the different populations.

The correlation analysis of the pairwise phenotypic traits showed strong positive correlation coefficients (r > 0.8, *p* ≤ 0.01), such as those of DBH and GD and among leaf dimensions, which aligned with fundamental biological expectations. Notably, the HWR showed a significant positive correlation with DBH and GD, implying that the heartwood production of the trees increased with an increase in DBH and GD. This correlation raises an intriguing question: does fast growth directly promote heartwood formation, or does it accelerate the senescence of central cells, thereby inducing heartwood development? This is also a question worth pondering and exploring. In any case, this result also implies that we need to integrate growth traits as secondary selection indices on heartwood yield in addition to anchoring the index of heartwood yield in the breeding process.

Association analysis is an analytical method that associates phenotypic traits with molecular markers with polymorphisms [39]. Owing to their high polymorphism, locus specificity, and genome-wide coverage, SSR markers have gained prominence in trait-associated marker studies across plant species. Representative applications include the following: *Perilla* [40], *Chenopodium quinoa Willd*. [41], *Pinus yunnanensis* [42], and *Elaeis guineensis Jacq.* [43].

Lander, E. [44] proposed that the results of association analysis are affected by kinship and population structure, and false positives may occur, resulting in pseudo-association between target genes and unrelated loci, so we employed a general linear model (GLM) and mixed linear model (MLM) in this study. The two models were used to analyze the association of nine phenotypic traits in *D. odorifera.* In the former, the Q matrix derived from the analysis of population structure was substituted into the association analysis for covariance to avoid false positives caused by the mixing of subgroups, and in the latter, a random effect Kinship matrix (i.e., Q + K) was introduced on top of the Q matrix to correct for the complex kinship relationships within the population and to reduce the false-positive rate of the results.

Population structure analysis was performed on 70 samples using STRUCTURE v2.3.2 software to derive the optimal subpopulation size K = 3. Linkage disequilibrium (LD) analysis is the genetic basis for association localization and informs the methodological strategy for association analysis. The LD results of this study showed that 97 pairs of paired loci with significant linkage disequilibrium were found, accounting for 35.14% of all locus combinations, and 168 pairs of paired loci with rD ≥ 0.1 (intermediate LD) accounted for 60.87% of all locus combinations under the threshold condition of *p ≤* 0.01. This widespread LD supports the validity of marker–trait association testing in this study.

Using Tassel v.5.0 software for correlation analysis, 24 pairs of SSR markers were correlated with nine traits. The results of the correlation analysis were as follows: we found that 34a-241, JXHT097-252, JXHT136-270, and S03-265 had highly significant correlations with the HWR, and the locus 96c-345 was only correlated with three traits, DBH, GD, and HWR, while the correlation with the other traits was not significant. Therefore, the four marker loci 34a-241, JXHT097-252, JXHT136-270, and S03-265 have certain potential to be applied in the future molecular marker-assisted breeding or prediction of yield traits of heartwood. In addition, 96c-345 not only indicates the HWR but also serves as a molecular marker for the assisted breeding of DBH and GD.

In short, in co-aged *D. odorifera* plantations, the significant positive correlations between the HWR and the growth traits DBH and GD highlight the need for a dual-trait selection strategy in breeding. In addition, the identification of four HWR-associated SSR loci and one multi-trait locus (96c-345) provides the molecular tools to implement this strategy, thereby directly contributing to marker-assisted breeding for enhanced heartwood yield.

While this study establishes a foundational framework, we acknowledge its limitations. Although this study did not cover all the locations of the current plantation forests, we obtained preliminary results showing that the genetic diversity of the current *D. odorifera* plantation has been well maintained, and future studies should expand the sampling locations for further exploration. In addition, this study also unearthed five SSR loci significantly associated with the phenotypic traits of *D. odorifera*, which provide initial targets for the marker-assisted selection (MAS) of *D. odorifera* and provide a reference for the excavation of loci associated with excellent traits of *D. odorifera* in the later stage. However, Rasheed et al. [45] have also suggested that although SSRs have been frequently used for gene localization and tagging, they have limited potential to be applied in practical applications due to allele scoring ambiguities, data incompatibility across platforms, biased genomic distribution, and low-throughput genotyping. Therefore, it is necessary to improve the sequencing of the *D. odorifera* genome, expand the sample size, and develop new molecular markers, such as a high-density SNP array, with higher polymorphism information content (*PIC*), more uniform distribution, and faster detection to achieve precise localization between markers and traits.

Based on these findings, we propose an integrated conservation–breeding strategy. The robust genetic diversity found in plantations provides a compelling mandate for establishing a germplasm bank, ensuring this variation is preserved for future generations. Concurrently, the transition from our foundational SSR markers to a high-density SNP array will empower a new era of precision breeding, directly translating these discoveries into tangible outcomes for growers. Ultimately, the success of these ex situ efforts depends on the establishment of natural reserves to protect the unique genetic identity of wild populations from genetic introgression. This tripartite approach—to conserve, utilize, and protect—offers a comprehensive roadmap to secure the genetic future of *D. odorifera* and serves as a model for other endangered timber species.

## 4. Materials and Methods

### 4.1. Plant Materials

All plant materials used in this study were collected from cultivated plantation populations, not from wild habitats. As shown in Table 7, a total of 380 *Dalbergia odorifera* T. Chen individuals were sampled from five populations across China: Dongfang (DF), Ledong (LD), Xinglong (XL), Haikou (HK) in Hainan Province, and Xishuangbanna (BN) in Yunnan Province. The climate across all sites is classified as tropical monsoon, with high annual temperatures (21–27 °C), concentrated rainfall during May-October (1000–2000 mm), and high relative humidity (75–85%). The soils are predominantly lateritic. This relatively consistent environmental background helps minimize the potential confounding effects of extreme ecological variation on the genetic parameters analyzed in this study.

From each individual, 15 fresh leaves were collected, immediately sealed in plastic bags with silica gel desiccant, and dried for subsequent DNA extraction. A subset of 70 trees from HK and XL underwent increment core extraction at breast height (1.3 m) for the quantification of the heartwood ratio (HWR). Additionally, 13 outgroup specimens (eight species of *Dalbergia* L. f. and five species of *Pterocarpus* Jacq.) were included to validate the species discrimination capacity of SSR markers, with all voucher specimens authenticated by Rongtao Li of the Hainan Branch Institute of Medicinal Plant Development, Chinese Academy of Medical Sciences.

### 4.2. Measurement of Phenotypic Traits

Phenotypic traits were quantified in 70 individuals from HK and XL, including diameter at breast height (DBH), ground diameter (GD), Rachis Length (RL), Leaflet Number (LN), Leaflet Length (LL), Leaflet Width (LW), Leaflet Length-to-Width Ratio (LWR), Leaflet Area (LA), and the heartwood ratio (HWR): heartwood length/debarked diameter in increment cores at DBH. These trait data served as phenotypic variables for SSR-based association mapping.

### 4.3. Genomic DNA Extraction and PCR Amplification

Genomic DNA was isolated from all 380 individuals using a Modified CTAB Plant DNA Kit (Aidlab Biotech, Beijing, China), with concentration and purity verified by NanoDrop 2000c (ThermoFisher, Waltham, MA, USA) spectrophotometry (OD260/280 ≥ 1.8) and integrity assessed via 1% agarose gel electrophoresis (80 V, 45 min). The amplification of 24 SSR loci was conducted in 10 μL reactions on Veriti™ 384-Well Thermal Cyclers (Applied BiosystemsFoster City, CA, USA) containing the following: 5.0 μL 2× Taq PCR Master Mix, 1.0 μL genomic DNA (20–50 ng/μL), 0.5 μL of each forward/reverse primer (10 pmol/μL), and 3.0 μL ddH_2_O. The thermocycling protocol included the following: Initial denaturation at 95 °C for 5 min; Touchdown phase consisting of 10 cycles of 95 °C for 30 s, annealing from 62 °C to 52 °C (−1 °C/cycle) for 30 s, 72 °C for 30 s; Amplification phase: 25 cycles of 95 °C for 30 s, 52 °C for 30 s, 72 °C for 30 s; Final extension: 72 °C for 20 min. PCR products were resolved by fluorescent capillary electrophoresis (ABI 3730xl, Applied Biosystems, Foster City, CA, USA) and genotyped using GeneMarker^®^ v2.7.0 to extract allele sizes, peak morphology, and biallelic genotypes.

### 4.4. Data Analysis

After exporting raw data from the ABI 3730xl instrument, GeneMarker^®^ v2.7.0 software [46] was used to read the genotyping data. Genotypic data were analyzed using GenAlEx v6.5 software [47] to estimate genetic diversity parameters: allele frequencies, observed and effective allele numbers (*N_a_* and *N_e_*), expected and observed heterozygosity (*H_e_* and *H_o_*), unbiased expected heterozygosity (*uHe*), Shannon’s Information Index (*I*), Wright’s fixation index (*F*), and F-statistics (including *H_S_*, *Fst*, *N_m_*, *G_st_*) [48,49,50]. Polymorphism information content (*PIC*) [51] per locus was calculated with CERVUS v3.0.7 [52]. GenAlEx v6.5 subsequently performed Hardy–Weinberg equilibrium tests, an analysis of molecular variance (AMOVA) [53], and principal coordinate analysis (PCoA). Phylogenetic reconstruction via UPGMA clustering (R v4.5.1) and population structure inference (STRUCTURE v2.3.4; K = 3–10, 20 replicates) [54,55] were conducted, with optimal K determined by the ΔK method (STRUCTURE HARVESTER v0.6.94).

For phenotypic traits, the minimum/maximum, range, mean, standard deviation (SD), and coefficient of variation (*CV*) were calculated per region and across all samples, followed by pairwise trait correlations and regional differentiation analysis. Association analysis employed PLINK v1.9 for linkage disequilibrium (LD) analysis and TASSEL v5.0 [56] for trait–marker associations under a general linear model (GLM) and mixed linear model (MLM), using population structure (*Q*) and kinship (*K*) matrices as covariates [57], with significance thresholds set at *p* ≤ 0.05 (significant) and *p* ≤ 0.01 (highly significant). Quantile–quantile (QQ) plots serve as a crucial tool for evaluating the efficiency of models used in association analysis in constructing population structures and identifying familial associations. These QQ plots were generated using the R package “CMplot”(https://github.com/YinLiLin/CMplot (accessed on 10 July 2025)) based on *p*-values from MLM and GLM analyses. The Manhattan scatter plot was constructed using the R package “qqman”(https://github.com/stephenturner/qqman (accessed on 10 July 2025)) [58].

## 5. Conclusions

This study provides compelling evidence for utilizing *Dalbergia odorifera* T. Chen plantations as sustainable genetic resources through multipopulation diversity assessment. A total of 380 individual SSR analyses of five populations showed that the genetic diversity of plantation forests (*I* = 1.0301; *H_o_* = 0.5175; *H_e_* = 0.5446; *PIC* = 0.4925) was at a medium level, comparable to wild stands, and the population differentiation was at a medium level (*F_st_* = 0.0611), confirming that plantations retain substantial wild genetic variation without bottleneck effects and could be used as an effective alternative resource to wild germplasm. Correlation analysis between the pairwise phenotypic traits revealed significant positive correlations (*p* ≤ 0.05) between diameter at breast height (DBH), ground diameter (GD), and heartwood ratio (HWR), suggesting that growth-related processes may be associated with heartwood formation and that the related phenotypes should be added as a basis for screening during the selection of high-yielding germplasm. In addition, the locus 96c-345 was significantly associated with DBH, GD, and HWR, which can be used as a marker for the early screening of fast-growing germplasm, and loci 34a-241, JXHT097-252, JXHT136-270, and S03-265 were also found to be highly significantly associated with the HWR only (*p* ≤ 0.01), laying a foundation for the molecular marker-aided breeding of high-yield heartwood in *D. odorifera* molecular marker-assisted breeding.

This study suggests that the conservation and breeding of *Dalbergia odorifera* can be revolutionized by leveraging its plantation resources. Our direct contribution is the provision of a molecular toolkit for marker-assisted selection, which enables the rapid breeding of elite cultivars with enhanced heartwood yield. Building on this finding, we propose a hierarchical strategy for resource sustainability: In the short term, our markers can be immediately deployed to select superior genotypes for establishing dedicated breeding orchards and for certifying the genetic identity of planting stock. In the medium term, the genetically diverse plantation populations identified herein should be prioritized as core collections for inclusion in germplasm banks. In the long term, these efforts must be coupled with the stringent in situ conservation of remnant wild trees to preserve the full spectrum of adaptive genetic variation. Our work thus bridges the gap between genetic resource conservation and practical breeding application.

## Figures and Tables

**Table 1:** Characterization of 24 SSR loci used in this study.

SSR Locus	Forward Sequence	Reverse Sequence	Allele Size (bp)	Reference
34a	GAAGCCATTGCTGTTGTGAA	ATGACCCAGAAGAGAAGCCA	254–285	Current study
S29	TCCCCGTTCCTCTCTCTCAG	GGACTGTCACATGGCACTCA	159–175	Liu F.M. et al., 2019 [13]
S04	GCTGTGGAGTCACGTTCTCA	TCCCCACAGAATCACAAGCC	300–345	Liu F.M. et al., 2019 [13]
S24	GCTGCAAATGCCAGTGCTTA	CGCTGTTGTCAGTGCATTGG	250–274	Liu F.M. et al., 2019 [13]
JXHT062	TCCCTATGGTCGATTTACAC	ACGGGTACCATACCCATATC	181–292	Xu, J. R. et al., 2024 [33]
58b	CTGCTAGTGCTCCCAGTTCC	AGCCAACGTAACGAACCAAC	160–180	Current study
S10	CACGTACCCAACCGTCAAGA	TCCGACGACCACCTAATCCT	282–294	Liu F.M. et al., 2019 [13]
JXHT097	ACGTAGCCAACAACAACCCA	ACCGAACAGAGCTGGAAAAG	241–272	Xu, J. R. et al., 2024 [33]
S01	AGTCCCGCCCACAAAATCAT	CTGGTCAGTCATTCCCCCAC	241–279	Liu F.M. et al., 2019 [13]
33c	TTAGGGATTTGTAGTCGCGG	TCGTGCTCTTCACATTCTGG	262–281	Current study
JXHT010	CGTCAACAAAGGCCACACTG	AGAGAGGCCCAACGACTTTG	225–258	Xu, J. R. et al., 2024 [33]
S11	AAAAAGCGAGGACTACGGCA	TGGAGAAGCAGTGCTCGTTT	246–258	Liu F.M. et al., 2019 [13]
S08	GGAAGAGAAATGGAGGGTAGCT	TGCCAGACAACCAGAATGCT	317–339	Liu F.M. et al., 2019 [13]
JXHT022	CCACATCCATCACCAAACGC	GGGAACTCGGTGTCCATTGA	241–281	Xu, J. R. et al., 2024 [33]
JXHT025	ACAAATCCACGGCTAGGCAA	TCTCCTCTCGTGGTACTCCG	157–267	Xu, J. R. et al., 2024 [33]
S09	ACCCTCCTCCTCCACCTTTT	ACCGGCTTCAGTGATTGGTT	245–292	Liu F.M. et al., 2019 [13]
JXHT081	AGGTTTCTTCGAGGCATAGG	CAGCAGCTGCTATTGAAACG	173–258	Xu, J. R. et al., 2024 [33]
JXHT129	CTCAGAGTCAGACCAACGCA	GAAGGTAAACAGCACGTGCC	246–258	Xu, J. R. et al., 2024 [33]
JXHT100	GATGTGGTGCCGTGCTACTC	GGCCTGAATCATTAGCCCCA	333–347	Xu, J. R. et al., 2024 [33]
S03	GCACGTGGTCAAAGCAATCA	ATGAGCCCCTTCTGCACTTC	157–286	Liu F.M. et al., 2019 [13]
96c	TGTTTACTCGCAGGCTTCCT	CGACGAATTGATCGAAGTGA	350–399	Current study
JXHT004	CAGTTAGGCTGGCGAGAGAG	CATCTACAGGGCATCCGGTC	246–292	Xu, J. R. et al., 2024 [33]
JXHT013	GCCGACCAAAGTATCACCCA	AGAAGACTGCTCTTGCCGAC	265–304	Xu, J. R. et al., 2024 [33]
JXHT136	ATTCGAGCTGAACGACGTCG	AACATTACAGTGCGCCTTGC	259–290	Xu, J. R. et al., 2024 [33]

**Table 2:** Genetic diversity statistics of 24 SSR loci across 380 *D. odorifera* individuals.

Locus	N	*N_a_*	*N_e_*	*I*	*H_o_*	*H_e_*	*F*	*H_s_*	*PIC*	*F_st_*	*N_m_*	*G_st_*
JXHT013	378	4	2.329	0.970	0.585	0.571	−0.025	0.743	0.508	0.114	1.949	0.107
JXHT129	378	5	3.349	1.294	0.712	0.701	−0.015	0.837	0.650	0.019	12.642	0.012
JXHT025	377	5	2.116	0.887	0.520	0.527	0.014	0.745	0.442	0.049	4.874	0.041
JXHT010	379	6	1.391	0.637	0.274	0.281	0.024	0.639	0.269	0.051	4.657	0.043
S10	380	3	1.693	0.708	0.413	0.409	−0.010	0.696	0.359	0.022	10.988	0.014
JXHT004	379	6	2.813	1.256	0.649	0.645	−0.007	0.807	0.600	0.018	13.833	0.010
S03	380	2	1.623	0.572	0.376	0.384	0.020	0.694	0.310	0.041	5.810	0.034
JXHT097	379	9	4.797	1.705	0.707	0.792	0.107	0.810	0.761	0.212	0.931	0.205
S04	379	6	2.262	1.054	0.377	0.558	0.324	0.742	0.510	0.078	2.964	0.068
S24	378	3	2.361	0.972	0.540	0.577	0.064	0.753	0.512	0.088	2.576	0.081
S29	380	4	2.109	0.911	0.558	0.526	−0.061	0.736	0.453	0.030	8.083	0.022
S01	377	4	1.655	0.709	0.371	0.396	0.062	0.710	0.352	0.021	11.639	0.012
S08	374	4	1.768	0.794	0.412	0.434	0.052	0.696	0.387	0.013	19.458	0.004
34a	369	8	3.050	1.440	0.631	0.672	0.061	0.831	0.638	0.018	13.806	0.009
58b	378	6	1.718	0.841	0.415	0.418	0.006	0.720	0.389	0.034	7.010	0.027
JXHT022	373	2	1.564	0.546	0.354	0.361	0.019	0.689	0.296	0.076	3.050	0.068
JXHT100	372	6	2.061	1.059	0.516	0.515	−0.002	0.731	0.484	0.043	5.514	0.036
JXHT136	365	12	6.543	2.023	0.570	0.847	0.327	0.884	0.829	0.076	3.060	0.065
JXHT081	376	6	2.499	1.157	0.630	0.600	−0.051	0.786	0.539	0.055	4.259	0.048
S09	375	6	2.838	1.184	0.667	0.648	−0.029	0.788	0.586	0.100	2.256	0.093
96c	378	9	3.174	1.473	0.653	0.685	0.046	0.798	0.650	0.051	4.646	0.043
33c	377	6	3.189	1.259	0.658	0.686	0.042	0.822	0.624	0.076	3.031	0.069
JXHT062	378	4	1.733	0.665	0.418	0.423	0.011	0.689	0.343	0.073	3.174	0.065
S11	377	2	1.710	0.606	0.414	0.415	0.003	0.695	0.329	0.109	2.043	0.102
Mean	376.5	5.3	2.5144	1.0301	0.5175	0.5446	0.0409	0.7517	0.4925	0.0611	6.3438	0.0532

Note: N: number of effectively genotyped individuals per locus; *N_a_*: observed allele number; *N_e_*: effective allele number; *I*: Shannon’s diversity index; *H_o_*: observed heterozygosity; *H_e_*: expected heterozygosity; *F*: fixation index; *H_s_*: intrapopulation gene diversity; *PIC*: polymorphism information content; *F_st_*: genetic differentiation coefficient; *N_m_*: gene flow, *N_m_* = [(1/*F_st_*) − 1]/4; *G_st_*: genetic differentiation coefficient.

**Table 3:** Genetic diversity statistics of *D. odorifera* populations across five geographical locations.

Pop	N	*Na*	*Ne*	*I*	*Ho*	*He*	*uHe*	*F*
BN	197.8	4.458	2.519	0.998	0.557	0.540	0.542	−0.034
DF	48.3	4.167	2.278	0.952	0.466	0.518	0.523	0.081
HK	35.0	3.458	1.888	0.734	0.354	0.402	0.408	0.098
XL	36.0	3.833	2.303	0.948	0.520	0.529	0.537	0.034
LD	59.4	4.333	2.290	0.961	0.524	0.527	0.532	0.007
Mean	75.30	4.0500	2.2556	0.9187	0.4840	0.5035	0.5084	0.0372

Note: Five populations (Pop) across China: Dongfang (DF), Ledong (LD), Xinglong (XL), Haikou (HK) in Hainan Province, and Xishuangbanna (BN) in Yunnan Province; N: Number of effectively genotyped individuals per locus; *N_a_*: Observed allele number; *N_e_*: Effective allele number; *I*: Shannon’s diversity index; *H_o_*: Observed heterozygosity; *H_e_*: Expected heterozygosity; *uHe*: Unbiased expected heterozygosity; *F*: Fixation index.

**Table 4:** Molecular analysis of variance (AMOVA) based on SSR markers on germplasm from 380 *D. odorifera* individuals.

Source	df	SS	MS	Est. Var.	%
Among Pops	4	210.857	52.714	0.364	5.39
Among Indiv	375	2484.940	6.627	0.238	3.52
Within Indiv	380	2337.500	6.151	6.151	91.09
Total	759	5033.297		6.753	100.00

Note: df: Degrees of freedom; SS: Sum of squares; MS: Mean of squares; Est. Var.: Estimate of variance; %: Percentage of total variation.

**Table 5:** Descriptive statistics of 70 *D. odorifera* individuals for traits under study.

Trait	Min	Max	Range	Mean	SD	*CV* (%)
DBH (cm)	8.70	20.35	11.65	14.13	2.32	16.43
GD (cm)	9.90	23.50	13.60	16.34	2.64	16.15
RL (cm)	11.23	24.93	13.70	16.34	3.17	19.40
LN	8.33	15.00	6.67	12.10	1.38	11.40
LL (cm)	49.24	100.89	51.65	72.32	13.44	18.59
LW (cm)	19.25	46.41	27.15	31.66	5.62	17.75
LWR	1.84	3.05	1.21	2.31	0.27	11.58
LA (cm^2^)	669.76	2867.27	2197.51	1617.38	544.91	33.69
HWR (%)	0.00	51.52	51.52	22.26	13.36	60.04

Note: Min: Minimum; Max: Maximum; SD: Standard Deviation; *CV*: Coefficient of Variation; DBH: Diameter at Breast Height; GD: Ground Diameter; RL: Rachis Length; LN: Leaf Number; LL: Leaf Length; LW: Leaf Width; LWR: Length–Width Ratio; LA: Leaf Area; HWR: Heartwood Ratio.

**Table 7:** Locations of five sampling populations of *D. odorifera.*

Location	Code	Longitude	Latitude	Samples
Xishuangbanna, Yunnan Province, China	BN	100.89° E	22.11° N	199
Dongfang, Hainan Province, China	DF	110.72° E	18.49° N	50
Xinglong, Hainan Province, China	XL	110.19° E	18.73° N	36
Ledong, Hainan Province, China	LD	108.90° E	18.52° N	60
Haikou, Hainan Province, China	HK	110.25° E	19.99° N	35

## Data Availability

All data generated or analyzed during this study are included in this published article and its Supplementary Materials files.

## Supplementary Materials

The following supporting information can be downloaded at https://www.mdpi.com/article/10.3390/plants14243787/s1, Figure S1: Differentiation analysis across populations (Nei’ genetic distance and Nei’ genetic identity); Table S1: Cross-amplification of 24 pairs of polymorphic SSR primers in eight species of *Dalbergia* L. f. and five species of *Pterocarpus* Jacq.; Table S2: Descriptive statistics of *D. odorifera* of two populations (HK and XL) for traits under study.
