# Supplementary material for: Accelerating Dalbergia odorifera Plantation Breeding: SSR-Based Genetic Diversity and Trait Associations for Enhanced Heartwood Yield
Source: Plants (Basel). 2025 Dec 12;14(24):3787. doi: 10.3390/plants14243787 (PMC12736494; doi:10.3390/plants14243787)
Supplement: Supplementary file 1 [file plants-14-03787-s001.zip › plants-3962975-supplementary.pdf]

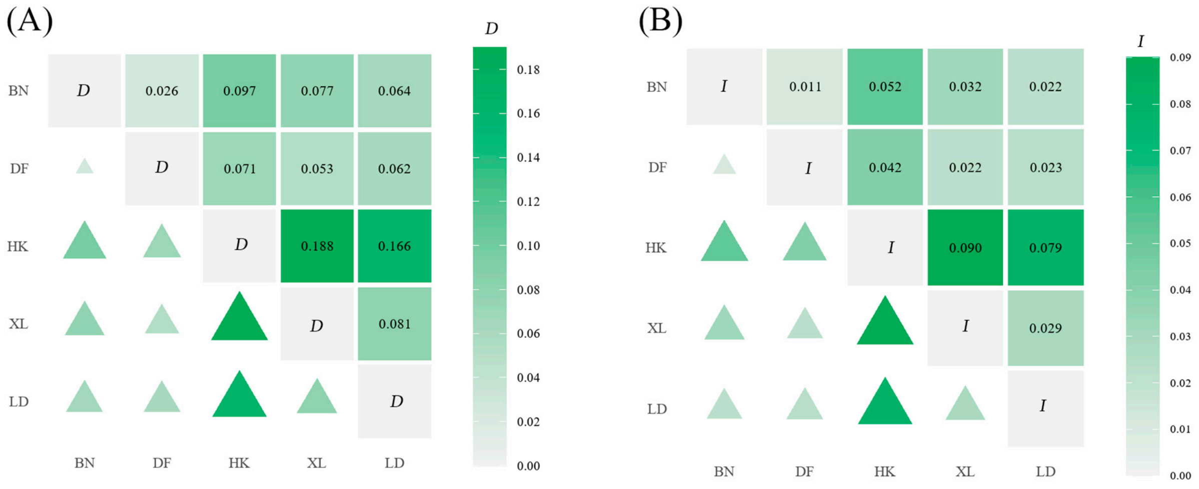

Figure S1 Genetic diversity across populations.

(A) Pairwise Nei' genetic distance (*D*) among five populations; (B) Pairwise Nei' genetic identity (*I*) among five populations.

Table S1 Cross-amplification of 24 pairs of polymorphic SSR primers in eight species of *Dalbergia* L. f. and five species of *Pterocarpus* Jacq.

locus	HT-1	HT-2	HT-3	HT-4	HT-5	HT-6	HT-7	HT-8	ZT-1	*ZT-2*	ZT-3	ZT-4	ZT-5	Amplification success rate in *Dalbergia* L. f. (%)	Amplification success rate in *Pterocarpus* Jacq. (%)
JXHT013	+	+	+	+	+	+	+	+	-	+	-	-	-	100	20
JXHT129	+	+	+	+	+	+	-	+	-	+	-	-	-	100	0
JXHT025	+	+	-	+	-	+	+	+	+	+	+	+	+	75	100
JXHT010	+	+	+	+	+	+	+	+	+	+	-	-	+	100	60
S10	-	-	+	+	+	+	-	-	-	-	-	-	-	50	0
JXHT004	+	+	+	+	+	+	+	+	+	+	+	+	+	100	100
S03	+	+	+	+	+	+	+	+	-	-	-	-	-	100	0
JXHT097	+	+	+	+	+	+	+	+	+	+	+	+	-	100	80
S04	+	+	+	+	+	+	+	+	+	+	+	+	+	100	100
S24	+	+	+	-	+	+	+	+	-	-	-	-	-	87.5	0
S29	-	+	+	+	+	+	+	+	-	+	+	-	-	87.5	40
S01	+	+	+	+	+	+	+	+	-	-	-	-	-	100	0
S08	-	-	+	+	+	+	-	-	-	-	-	-	-	50	0
34a	+	+	+	+	+	+	+	+	-	-	-	-	-	100	0
58b	+	+	+	-	+	+	-	+	-	-	-	+	-	75	20
JXHT022	-	-	-	+	+	+	+	-	-	-	-	-	-	50	0
JXHT100	+	+	+	+	+	+	+	+	+	+	+	-	+	100	80
JXHT136	-	-	-	-	+	-	-	-	-	-	-	-	-	12.5	0
JXHT081	+	+	+	+	+	+	+	+	-	+	+	-	-	100	40
S09	-	+	+	+	+	+	-	+	-	+	-	+	-	75	40
96c	-	-	+	-	+	-	+	+	+	+	+	+	+	50	100
33c	+	+	+	+	+	+	+	+	-	-	-	-	-	100	0
JXHT062	-	+	-	-	-	-	-	-	-	-	+	-	-	12.5	20

locus	HT-1	HT-2	HT-3	HT-4	HT-5	HT-6	HT-7	HT-8	ZT-1	*ZT-2*	ZT-3	ZT-4	ZT-5	Amplification success rate in *Dalbergia* L. f. (%)	Amplification success rate in *Pterocarpus* Jacq. (%)
S11	+	+	+	+	+	+	+	+	-	-	-	-	-	100	0

Note: HT-1: *Dalbergia oliveri* Gamble ex Prain; HT-2: *Dalbergia bariensis* Pierre; HT-3: *Dalbergia cultrate* Graham ex Benth.; HT-4: *Dalbergia benthamii* Prain; HT-5: *Dalbergia sissoo* Roxb. ex DC.; HT-6: *Dalbergia cochinchinensis* Laness; HT-7: *Dalbergia balansae* Prain; HT-8: *Dalbergia hainanensis* Merr. & Chun; ZT-1: *Pterocarpus marsupium* Roxb; ZT-2: *Pterocarpus macrocarpus* Kurz; ZT-3: *Pterocarpus septentrionalis* Donn. Smith; ZT-4: *Pterocarpus indicus* Wall.; ZT-5: *Pterocarpus echinatus* Pers. ' + ' indicates that the SSR loci were all successfully transferred and ' - ' indicates that the SSR loci were not amplified successfully.

Table S2 Descriptive statistics of *D. odorifera*. Genotypes of 2 Populations for the traits under study

Trait	Pops	Min	Max	Range	Mean	SD	CV(%)
DBH (cm)	XL	9.90	20.35	10.45	14.46	2.41	16.66
	HK	8.70	18.60	9.90	13.79	2.18	15.80
GD (cm)	XL	12.30	23.50	11.20	16.76	2.75	16.43
	HK	9.90	22.00	12.10	15.91	2.45	15.38
RL (cm)	XL	12.37	24.93	12.57	18.21	2.80	15.38
	HK	11.23	19.53	8.30	14.35	2.17	15.12
LN	XL	10.33	14.67	4.33	12.30	0.99	8.05
	HK	8.33	15.00	6.67	11.89	1.67	14.06
LL (cm)	XL	50.89	91.90	41.01	68.40	11.17	16.33
	HK	49.24	100.89	51.65	76.48	14.37	18.78
LW (cm)	XL	22.67	45.31	22.64	31.87	5.34	16.76
	HK	19.25	46.41	27.15	31.44	5.89	18.74
LWR	XL	1.84	2.61	0.77	2.17	0.19	8.62
	HK	1.99	3.05	1.06	2.46	0.26	10.45
LA (cm^3)	XL	810.66	2822.67	2012.01	1519.94	474.78	31.24
	HK	669.76	2867.27	2197.51	1720.73	593.32	34.48
HWR（%）	XL	0.00	44.07	44.07	21.15	11.65	55.06
	HK	0.00	51.52	51.52	23.36	14.80	63.35

Note: Min: Minimum; Max: Maximum; SD: Standard Deviation; CV: Coefficient of Variation; DBH: Diameter at Breast Height; GD: Ground Diameter; RL: Rachis Length; LN: Leaf Number; LL: Leaf Length; LW: Leaf Width; LWR: Length-width Ratio; LA: Leaf Area; HWR: Heartwood Ratio.
